# Neoadjuvant chemotherapy and radiotherapy outcomes in borderline‐resectable and locally‐advanced pancreatic cancer patients

**DOI:** 10.1002/cam4.5523

**Published:** 2022-12-07

**Authors:** Gregory P. Botta, Tridu R. Huynh, Samantha R. Spierling‐Bagsic, Alexander Agelidis, Randolph Schaffer, Ray Lin, Darren Sigal

**Affiliations:** ^1^ Division of Hematology/Oncology, Department of Medicine, Moores Cancer Center University of California San Diego La Jolla California USA; ^2^ Division of Medical Oncology Scripps MD Anderson Cancer Center La Jolla California USA; ^3^ Scripps Research Translational Institute La Jolla California USA; ^4^ Division of Internal Medicine Scripps Clinic/Green Hospital La Jolla California USA; ^5^ Scripps Whittier Diabetes Institute Scripps Health San Diego California USA; ^6^ Division of Hepatopancreatobiliary Surgery Scripps MD Anderson Cancer Center La Jolla California USA; ^7^ Division of Radiation Oncology Scripps MD Anderson Cancer Center La Jolla California USA

**Keywords:** chemotherapy, pancreatic cancer, radiation, resection

## Abstract

**Background:**

There is no agreed upon standard of care for borderline‐resectable pancreatic cancer (BRPC) or locally‐advanced pancreatic cancer (LAPC) patients regarding the benefit of chemotherapy or radiation alone or in combination.

**Patients and Methods:**

We completed a retrospective cohort analysis of BRPC and LAPC patients at a cancer center with expertise in multi‐disciplinary pancreatic ductal adenocarcinoma (PDAC) treatment over a 5‐year period from 03/01/2014 to 03/01/2019 (cut‐off date). The total evaluable newly diagnosed, treatment naïve, BRPC, and LAPC patients with adequate organ function and ability to obtain treatment after multidisciplinary review was 52 patients. After analysis, patients were evaluated for rates of resection, extent of resection (R0 or R1), median progression‐free survival (mPFS), and median overall survival (mOS).

**Results:**

Patients were treated with chemotherapy alone (gemcitabine and nab‐paclitaxel = 77% (20/26); FOLFIRINOX = 19% (5/26); single agent gemcitabine 3.8% (1/26)), or chemotherapy followed by chemoradiation (gemcitabine +5 Gy × 5 weeks), or chemoradiation alone prior to re‐staging and potential resection. Of the 29% (15/52) of patients who went on to surgical resection, 73% (11/15) achieved R0 resection. An R0 resection was achieved in 35% (9/26) of patients treated with chemotherapy alone, 7.6% (1/13) in a patient treated with chemotherapy followed by radiation, and 7.6% (1/13) with concurrent chemoradiotherapy alone. Chemotherapy alone achieved a mPFS of 16.4 months (*p*  < 0.0025) and mOS of 26.2 months (*p*  < 0.0001), chemotherapy followed by chemoradiation was 13.0 months and 14.9 months respectively, while concurrent chemoradiotherapy was 6.9 months and 7.3 months.

**Conclusions and Relevance:**

BRPC and LAPC patients capable of surgery after only receiving neoadjuvant treatment with chemotherapy had higher rates of R0 resection with prolonged median PFS and OS compared with any patient needing combination chemotherapy with radiotherapy.

## Graphical abstract

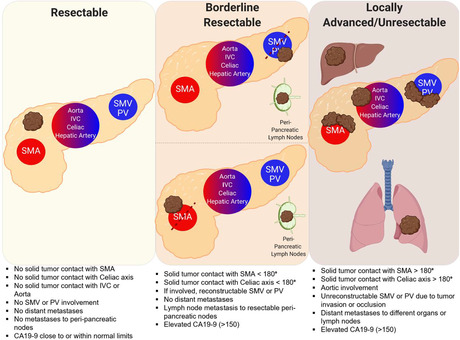

## 1 INTRODUCTION

**FIGURE 1:**
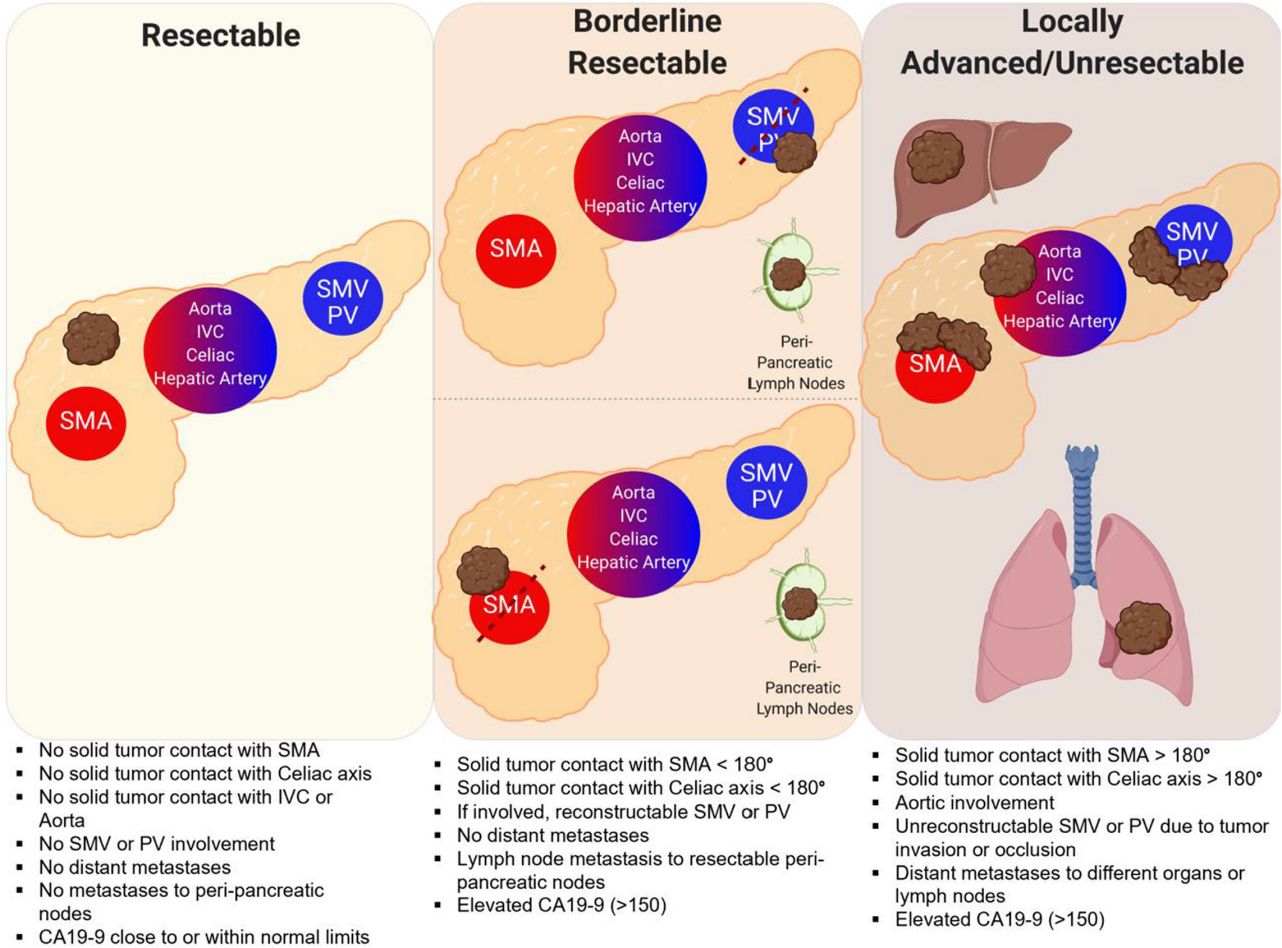
Pancreatic cancer resectability diagram. The surgical resectability of pancreatic adenocarcinoma falls on a spectrum between resectable to borderline to unresectable. Each patient's anatomy, disease progress, and blood vessel involvement must be individualized and discussed in a multidisciplinary tumor board before resection can be determined. Specialized surgical centers are capable of advanced resection and blood vessel grafting techniques in coordination with vascular surgery. Abbreviations: CA19‐9, Cancer Antigen 19–9; IVC, inferior vena cava; SMA, superior mesenteric artery; SMV, superior mesenteric vein; PV, portal vein.

Despite continued advances in the diagnosis and treatment of pancreatic cancer, it remains one of the most lethal malignancies, with an all‐stage 5‐year overall survival (OS) rate of approximately 10%.^1^ Pancreatic ductal adenocarcinoma (PDAC) constitutes the major histologic subset of resected pancreatic neoplasms (85–90%).^2^ Unfortunately, due to low‐rates of early detection, 80–85% of PDAC are diagnosed with metastatic or unresectable locally‐advanced disease, leaving only the remaining 15–20% of patients as possible candidates for surgical resection—the only potentially curative option.^3^ The definition of resectable PDAC falls along a continuum and can include non‐metastatic, borderline‐resectable pancreatic cancer (BRPC), but usually excludes locally‐advanced pancreatic cancer (LAPC) (Figure 1). A PDAC that is immediately resectable has a high likelihood of a margin‐negative (R0) resection. Other criteria considered to determine resection candidacy include CA19‐9 level, with high levels (>150) seen as a deterrent for upfront resection and necessitating normalization by neoadjuvant therapy prior to potential surgery.^4^ Borderline‐resectable PDAC has anatomic intimacy with vessels such that a residual microscopic‐positive (R1) resection will likely ensue. LAPC is defined as a lesion with an indeterminate likelihood of leading to a margin‐negative (R0) resection based, predominantly, on the degree of arterio‐venous involvement and with high chance of gross margin‐positive disease (R2).^5^, ^6^

Current guidelines from the National Comprehensive Cancer Network (NCCN) and American Society of Clinical Oncology (ASCO) recommend neoadjuvant therapy followed by restaging and resection in eligible patients.^7^, ^8^ This is based on nearly two decades of largely retrospective studies which have shown an overall benefit for neoadjuvant therapy in BRPC, with higher rates of R0 resections and OS.^9^, ^10^, ^11^, ^12^, ^13^, ^14^, ^15^, ^16^, ^17^, ^18^ In the locally‐advanced setting, various trials have analyzed chemotherapy alone versus concurrent chemoradiation versus radiation alone. The SCALOP trial evaluating concurrent chemoradiation with capecitabine versus gemcitabine observed that capecitabine was superior in terms of mOS.^19^ The superiority of chemotherapy alone was thought to have been confirmed by the FFCD‐SFRO study, which observed that single agent gemcitabine alone was superior to concurrent fluorouracil and cisplatin with radiation in terms of overall survival.^20^ Oppositely, however, ECOG‐4201 demonstrated improved overall survival with gemcitabine and concurrent radiation over gemcitabine monotherapy.^21^ The LAP07 trial evaluated gemcitabine with or without the epidermal growth factor receptor (EGFR) inhibitor erlotinib versus concurrent chemoradiation with capecitabine.^22^ Although no difference in median overall survival was found, local recurrence was significantly less with the addition of radiation. Recently, the PREOPANC trial evaluated immediately resectable or BRPC patients receiving pre‐operative chemoradiotherapy with gemcitabine compared with those receiving only surgery. The preoperative chemoradiation group was shown to have higher rates of R0 resection, improved disease‐free as well as locoregional‐failure free survival when compared with immediate surgery. There was, however, no difference in median overall survival between the two groups.^17^ In 2016, the A021101 trial evaluated patients with borderline PDAC treated with FOLFIRINOX for 2 months followed by capecitabine plus 50.4 Gy radiation.^23^ Those deemed able to proceed with surgery did so. The study showed that 68% of borderline‐resectable PDAC patients were able to get to resection with 93% having an R0 resection and 13% having a complete pathologic response. The median overall survival was 21.7 months. Recently, the Alliance A021501 study was completed evaluating preoperative FOLFIRINOX versus FOLFIRINOX with radiation therapy (RT) (either stereotactic body RT 33–40 Gy in 5 fractions or hypofractionated image guided RT 25 Gy in 5 fractions) in borderline‐resectable pancreatic cancer. The study was not statistically developed to compare the two arms. Nonetheless, the 18 months overall survival (OS) rate in the chemotherapy alone group was 67.9% while the chemoradiation group was 47.3%. At 31 months, the median OS was 31% in the chemotherapy alone group and 17.1% in the chemoradiation group. Interestingly, RT with chemotherapy did not seem to improve OS compared to chemotherapy alone or historical data; albeit these patients had a higher median CA19‐9 level during randomization.^24^

As is expected with multiple trials showing conflicting data, there is currently no agreed upon standard or high‐level evidence regarding what may be an optimal neoadjuvant regimen in BRPC or LAPC to maximize an attempt at R0 resection. This in turn has led to a plethora of trials in this space with a variety of treatments including FOLFIRINOX^25^, ^26^; FOLFIRINOX followed by capecitabine and radiotherapy^27^; FOLFIRINOX with losartan^28^; cisplatin, epirubicin, capecitabine and gemcitabine (PEXG)^16^; gemcitabine, S‐1, and leucovorin (GSL)^29^; S‐1 with concurrent hypofractionated radiotherapy^30^; or chemoradiation with gemcitabine.^31^

In this single‐center retrospective cohort study, we report our experience with BRPC and LAPC patients treated with chemotherapy alone, chemotherapy followed by chemoradiation, or concurrent chemoradiation alone.

## 2 METHODS

### 2.1 Study population

**FIGURE 2:**
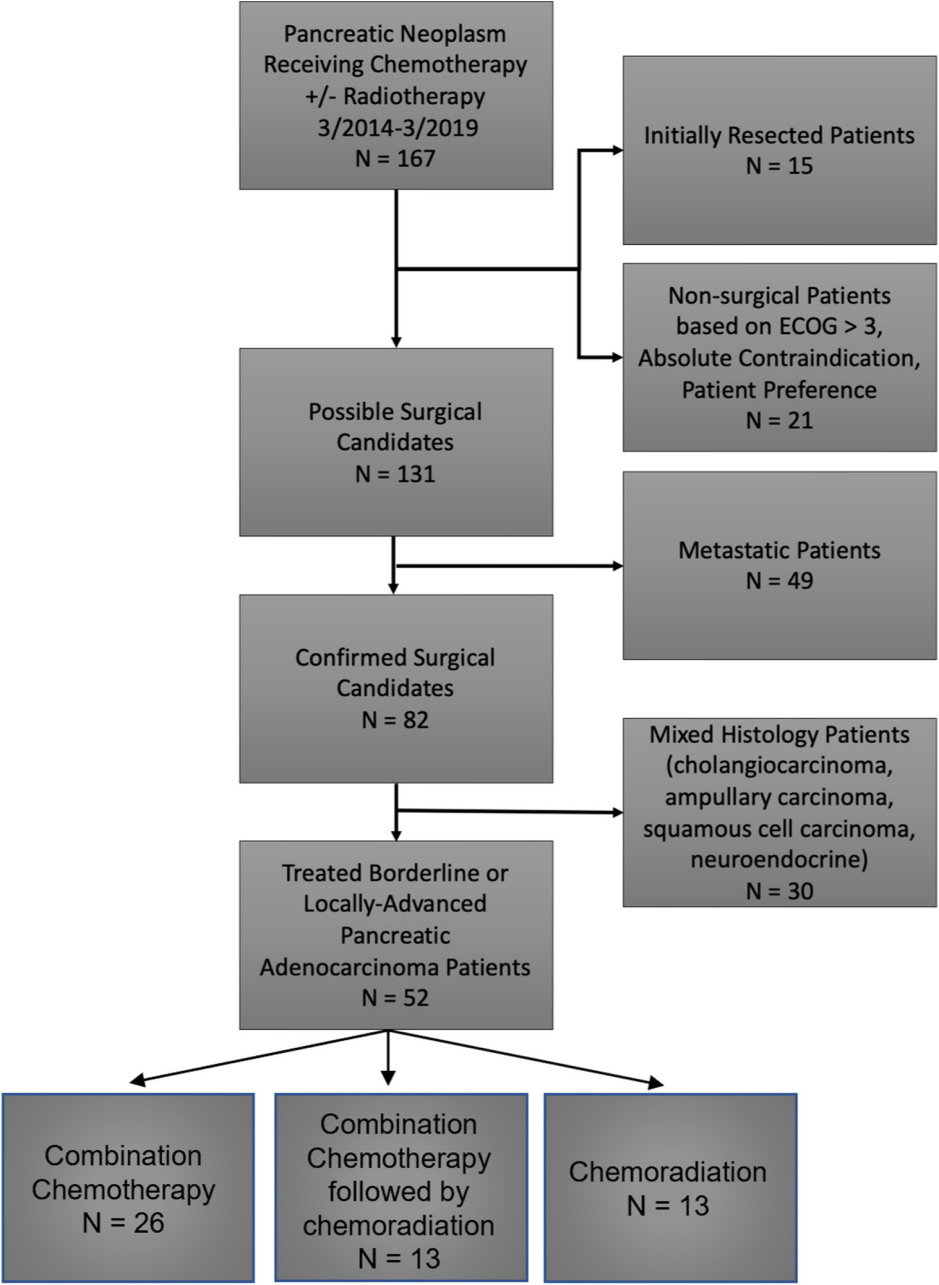
Study flowchart of resectable pancreatic ductal adenocarcinoma patients receiving chemotherapy +/− radiotherapy. Data extracted from electronic medical record system. Non‐surgical patients were determined by extensive chart review and metastatic patients were removed based upon staging and imaging review. Those patients with confirmed pathology showing a mixed histology were also removed such that only pancreatic adenocarcinoma patients were evaluated.

The study population was acquired through a retrospective analysis of the electronic medical record at Scripps MD Anderson Cancer Center. Institutional IRB approval was obtained for retrospective chart review (IRB 19–7331). Boolean search logic filtered for patients greater than 18 years old diagnosed with a pancreatic neoplasm (ICD‐10 codes C25.0, C25.1, C25.2, C25.3, C25.4, C25.7, C25.8, C25.9) who received chemotherapy with or without radiotherapy during the 5‐year period spanning 3/1/2014 to 3/1/2019 (cut‐off). The above search criteria returned a total of 167 patients (Figure 2). Of these, 21 patients were not surgical candidates due to poor performance status, medical contraindications, or personal preference after multidisciplinary review. Resectable patients that underwent upfront resection and no neoadjuvant therapy were excluded (15). De novo metastatic pancreatic cancer patients (49) were also not eligible. Cross‐coding of cholangiocarcinoma, ampullary adenocarcinoma, squamous cell carcinoma, or neuroendocrine neoplasm excluded an additional 30 patients. The remaining 52 patients were included in the final analysis as having borderline‐resectable (33/52, 63%) or locally‐advanced (19/52, 37%) pancreatic ductal neoplasms. All patients were biopsy confirmed pancreatic ductal adenocarcinoma prior to management decisions. Management of these patients was determined after evaluation by a multi‐disciplinary tumor board committee that included pancreatobiliary surgeons, gastrointestinal medical oncologists, radiation oncologists, pathologists, and radiologists. Recommendations consisted of either neoadjuvant chemotherapy (26/52, 50%), chemotherapy followed by chemoradiotherapy (13/52, 25%), or concurrent chemoradiotherapy (13/52, 25%) before restaging and determination of surgical candidacy. Radiation or chemotherapy was completed at a minimum of 28 days prior to resection.

Definition of borderline‐resectable and locally‐advanced pancreatic neoplasms were based on the NCCN criteria.^7^ Borderline‐resectable neoplasms were defined as tumor with abutment or short segment encasement of the common hepatic artery, or abutment of the superior mesenteric artery less than 180 degrees, or abutment or encasement of the superior mesenteric vein or portal vein deemed to be amenable to surgical reconstruction. Locally advanced neoplasms were defined as tumor with more than 180 degrees encasement of the superior mesenteric artery, any involvement of the celiac artery, and involvement of the superior mesenteric vein or portal vein deemed unamenable to surgical reconstruction (Figure 1). R0 resection was defined as negative presence of tumor cells at the ink margin.

Patients were treated with three to six cycles of chemotherapy (gemcitabine and nab‐paclitaxel, FOLFIRINOX, or gemcitabine alone), chemotherapy followed by chemoradiation (gemcitabine plus 5 Gy × 5 weeks), or concurrent chemoradiation alone prior to evaluation for resection by a multidisciplinary team after restaging scans. Patients then completed adjuvant chemotherapy per the treating oncologist. Each chemotherapy regimen was chosen based on the patient's performance status, pre‐existing co‐morbidities, and likely tolerability of the backbone sequence. For example, if a patient was not expected to tolerate FOLFIRINOX, they would be sequenced from gemcitabine and nab‐paclitaxel to 5‐fluorouracil + liposomal irinotecan. Otherwise, a sequence of FOLFIRINOX followed by gemcitabine and nab‐paclitaxel was considered.

The decision of each treatment pathway was based upon the weight of various factors including the patient's performance status, co‐morbidities, disease burden (number of clinically significant lymph nodes, tumor size), CA19‐9 level (above or below 150 U/ml), and anatomy (tumor location in the pancreas, blood vessel involvement). The bias was toward neoadjuvant combination chemotherapy where perceived as tolerable to the patient, especially for CA19‐9 over 150 U/ml. No patient was pre‐selected for chemotherapy followed by chemoradiation and only proceeded to chemoradiation when neoadjuvant chemotherapy was unable to bring the patient to resection at interval staging.

Each patient completed the multi‐disciplinary tumor board recommendation in terms of treatment prior to resection. Combination chemotherapy was at times delayed due to neutropenia or dose reduced for diarrhea or peripheral neuropathy. All patients who completed the radiation pathway tolerated radiation to the pre‐determined amount without early cessation.

## 3 STATISTICAL ANALYSIS

Clinical and demographic variables were compared between treatment regimens by Chi‐square test for categorical data or *t‐*tests for continuous data. CA19‐9 values before and after treatment were evaluated by Wilcoxon matched pairs signed‐rank test. Survival curves were generated using the Kaplan–Meier methods, and log‐rank methods were used for comparing curves and estimating hazards ratios and median survival. All analyses were conducted using GraphPad Prism v. 8.0.

## 4 RESULTS

### 4.1 Patient's characteristics

The mean age of the 52 patient cohort was 70.6 years (range of 44 to 87 years) (Table 1). Female patients made up 31/52 patients (59.6%). Eastern Cooperative Oncology Group (ECOG) performance status stratified as follows: 6/52 (11.5%) had an ECOG status of 0, 35/52 (67.3%) a status of 1, and 11/52 (21.2%) a status of 2. Regarding neoplasm resection classification, 33/52 (63.5%) were borderline‐resectable and 19/52 (36.5%) were locally‐advanced. Median CA19‐9 level at presentation was 717.5 U/mL (IQR 237.1–2651), prior to therapy was 555.1 (IQR 188.8–2766), and after therapy was 106.5 (IQR 35.65–559.77). Considering anatomical location, 38/52 (73.1%) of the tumors were located at the head of the pancreas, with the remaining 14/52 (26.9%) in the body or tail of the pancreas.

**TABLE 1:** Characteristics of 52 patients undergoing neoadjuvant combination chemotherapy, chemotherapy followed by chemoradiation, or concurrent chemoradiation

	All patients (*N* = 52)	Chemotherapy alone (*N* = 26)	Chemotherapy followed by chemoradiation (*N* = 13)	Concurrent chemoradiation (*N* = 13)
Age in years				
Mean (SD)	70.6 (7.8)	70 (7.6)	69 (2.0)	73 (2.4)
Range	44–87	44–82	55–77	60–87
Female, *N* (%)	31 (59.6)	10 (38.5)	10 (77)	9 (69)
ECOG, *N* (%)				
0	6 (11.5)	4 (15.4)	1 (7.7)	1 (8)
1	35 (67.3)	18 (69.2)	7 (54)	8 (61)
2	11 (21.2)	4 (15.4)	5 (38)	4 (31)
Stage, *N* (%)				
BR	33 (63.5)	18 (69.2)	7 (54)	8 (62)
LA	19 (36.5)	8 (30.8)	6 (46)	5 (38)
CA19‐9 U/ml (at presentation)				
Median	717.5	556	1615.8	691.6
IQR	237.1–2651	122.4–1763	307.4–3774.5	263–2651.4
CA19‐9 U/ml (pre‐therapy)				
Median	555.1	346	1433	461.2
IQR	188.8–2766	98.3–1621	487.6–3544.8	227.6–4205.15
CA19‐9 U/ml (post‐therapy)				
Median	106.5	52	159.9	612
IQR	35.65–559.77	37.6–571.82	73.1–660.5	234.8–2224.7
*p* value^a^	5.791 e‐6	2.050 e‐5	0.0039	0.3013
Tumor site, *N* (%)				
Head	38 (73.1)	23 (88.5)	7 (54)	8 (62)
Body/Tail	14 (26.9)	3 (11.5)	6 (46)	5 (38)
Tumor largest diameter (cm), mean (SD)		3.07 (1.35)	3.71 (0.25)	4.37 (0.57)
Rate of Resection R0, *N* (%)	11 (21)	9 (35)	1 (8)	1 (8)
R1, *N* (%)	4 (8)	4 (15)	0 (0)	0 (0)
Non‐resected, *N* (%)	37 (71)	13 (50)	12 (92)	12 (92)

Abbreviations: BR, borderline resectable, LA, locally advanced, SD, standard deviation, IQR, interquartile range, R0, microscopic tumor clearance, R1, microscopic tumor infiltration.

^a^ Wilcoxon matched pairs signed‐rank test. Student's *t*‐test was used for parametric variables.

In the neoadjuvant chemotherapy alone group, the mean age was 70 years old (range 44–82 years) (Table 1). Of these, 10/26 (38.5%) of the patients were female. In terms of ECOG status, 4/26 (15.4%) had an ECOG performance status of 0, 18/26 (69.2%) a status of 1, and 4/26 (15.4%) a status of 2. Borderline‐resectable patients amounted to 18/26 (69.2%) of the tumors, and 8/26 (30.8%) were locally‐advanced. Median CA19‐9 level at presentation was 556 (IQR 122.4–1763), prior to therapy 346 (IQR 98.3–1621), and post‐therapy 52 (IQR 37.6–571.82). Anatomically, 23/26 (88.5%) of the tumors were located at the head of the pancreas with the remaining 3/26 (11.5%) in the body or tail of the pancreas. The mean largest diameter of the tumors in that group was 3.07 cm.

In the chemotherapy followed by chemoradiation group, the mean age was 69 years (range of 55 to 77) (Table 1). Of these patients, 10/13 (77%) were female. ECOG performance status of 0 was 1/13 (8%), 7/13 (54%) had a status of 1, and 5/13 (38%) had a status of 2. Borderline‐resectable tumors made up 7/13 (54%) of the tumors and 6/13 (46%) were locally‐advanced. Median CA19‐9 level at presentation was 1615.8 (IQR 307.4–3774.5), prior to therapy was 1433 (IQR 487.6–3544.8), and post therapy was 159.9 (IQR 73.1–660.5). In terms of location, 7/13 (54%) of the tumors were located in the head of the pancreas, with the remaining 6/13 (46%) located in the body or tail of the pancreas. The mean largest diameter of the tumors was 3.71 cm in that group.

Lastly, in the concurrent chemoradiation group, the mean age was 73 years (range of 60–87) (Table 1). Of these patients, 9/13 (69%) were female. ECOG performance status of 0 was 1/13 (8%), 8/13 (61%) had a status of 1, and 4/13 (31%) had a status of 2. Borderline‐resectable tumors made up 8/13 (62%) of the tumors and 5/13 (38%) were locally‐advanced. Median CA19‐9 level at presentation was 691.6 (IQR 263–2651.4), prior to therapy was 461.2 (IQR 227.6–4205.2), and post therapy it was 612 (IQR 234.8–2224.7). In terms of location, 8/13 (62%) of the tumors were located in the head of the pancreas, with the remaining 5/13 (38%) located in the body or tail of the pancreas. The mean largest diameter of the tumors was 4.37 cm in that group.

### 4.2 Chemotherapy regimen breakdown

In terms of all‐comers, the majority of patients received combination gemcitabine with nab‐paclitaxel as their chemotherapy (33/52 patients, 63%) (Table S1A:). The second most common chemotherapy was gemcitabine alone at 13/52 patients (25%). Only 6/52 (12%) patients received FOLFIRINOX as their neoadjuvant chemotherapy.

In the chemotherapy alone group, 20/26 (77%) of the patients received gemcitabine with nab‐paclitaxel, 5/26 (19%) received FOLFIRINOX, and 1/26 (3.8%) received gemcitabine alone as their neoadjuvant chemotherapy regimen.

In the chemotherapy followed by chemoradiation group, 13/26 (50%) of the patients received gemcitabine with nab‐paclitaxel, 1/26 (3.8%) received FOLFIRINOX, and 12/26 (46.2%) received gemcitabine alone as their neoadjuvant chemotherapy regimen. All patients received gemcitabine with their radiation (13/26 (50%) in the chemotherapy followed by chemoradiation group and 13/26 (50%) in the concurrent chemoradiation group).

### 4.3 Rates of R0, R1, and non‐resection

Out of all patients in this study, 15/52 (29%) patients underwent resection, with 11/15 (73%) having R0 resections after neoadjuvant therapy (Table 1). In the chemotherapy alone group, 13/26 (50%) patients underwent resection, with 9/13 (69%) being R0 resections. For the chemotherapy followed by chemoradiation group, 1/13 (8%) patients underwent surgery resulting in an R0 resection. Similarly, in the concurrent chemoradiation group, 1/13 (8%) patients underwent surgery resulting in an R0 resection.

Comparing resection rates between BRPC and LAPC, 13/33 (39%) of BRPC patients underwent resection, with 9/13 (69%) being R0 resections. For LAPC, 2/19 (10%) of patients underwent resections (both R0) (Table S1B).

### 4.4 Median overall survival and progression‐free survival

**FIGURE 3:**
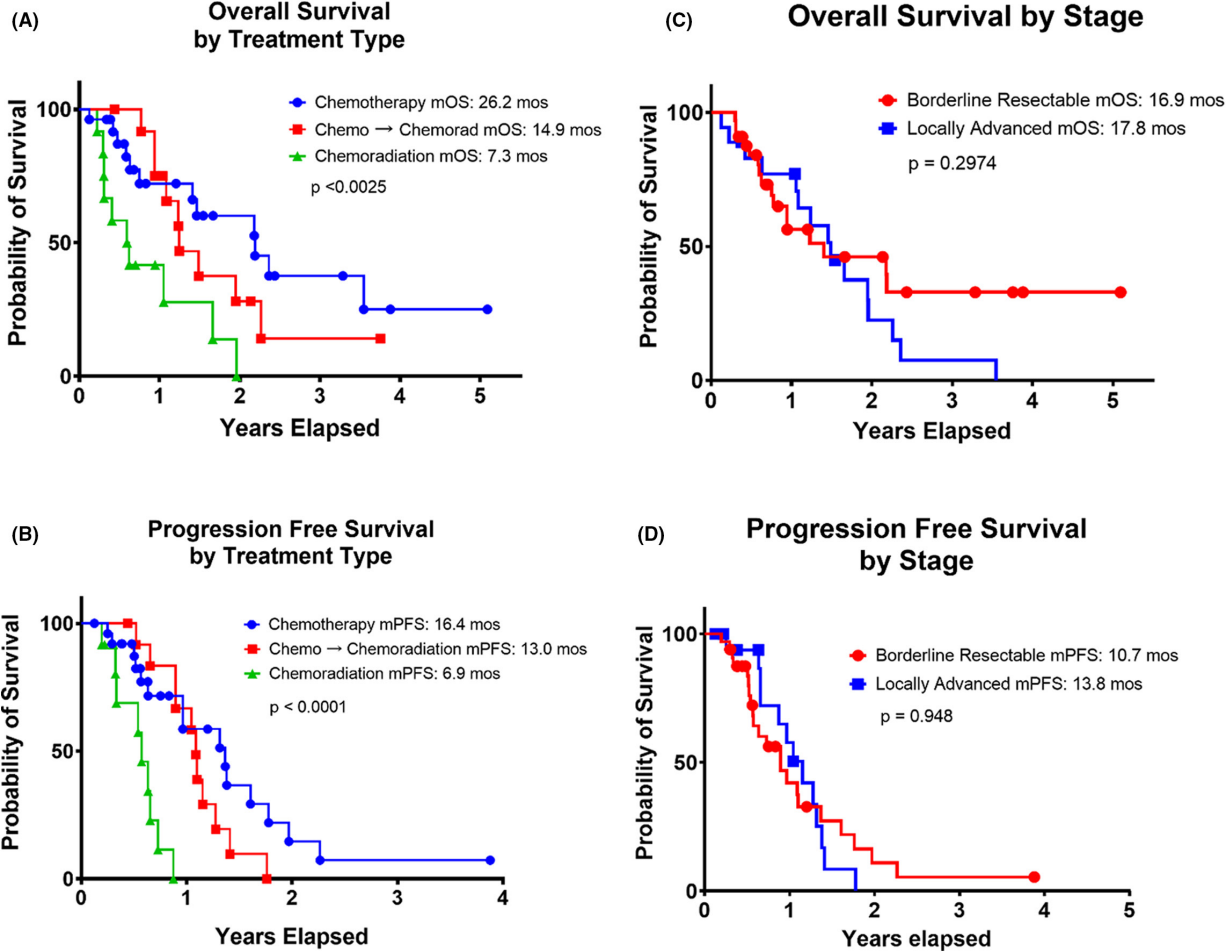
Kaplan–Meier Analysis of Resectable and Borderline‐resectable Pancreatic Cancer Patients by Treatment Type and Stage at diagnosis. Median overall survival (mOS) and median progression‐free survival (mPFS) comparing neoadjuvant chemotherapy, chemotherapy followed by chemoradiation, and chemoradition (A&B) in borderline resectable and locally advanced pancreatic cancers (C&D). Combination chemotherapy resulted in a mOS of 26.2 months, while chemotherapy followed by chemoradiaton resulted in a mOS of 14.9 months, and chemoradiation resulted in a mOS of 7.3 months (*p* < 0.0025). Median progression free survival (mPFS) for combination chemotherapy was 16.4 months compared to 13.0 months for chemotherapy followed by chemoradiation and 6.9 months for chemoradiation (*p* < 0.0001). mOS for borderline resectable patients was 16.9 months compared to 17.8 months for locally advanced (*p* = 0.2974). mPFS for borderline resectable was 10.7 months compared to 13.8 months for locally advanced (*p* = 0.948). *p*‐value determined by log rank.

The median overall survival (OS) was calculated from the date of diagnosis for all 52 patients and was 17.5 months while the median progression‐free survival (PFS) was 11.6 months. In the neoadjuvant chemotherapy alone group, median OS was 26.2 months, and the median PFS was 16.4 months. For the chemotherapy followed by chemoradiation group, median OS was 14.9 months, and the median PFS was 13.0 months. Lastly, for the concurrent chemoradiation group, median OS was 7.3 months, and the median PFS was 6.9 months. Statistical analysis of the Kaplan–Meier curves showed a statistical significance both in terms of median OS (*p* < 0.0025) (Figure 3A) and median PFS (*p* < 0.0001) (Figure 3B) between chemotherapy alone, chemotherapy followed by chemoradiation, and concurrent chemoradiation.

Comparing BRPC to LAPC regardless of treatment modality, BRPC patients had a median OS of 16.9 months compared with 17.8 months for LAPC. Median PFS for BRPC patients was 10.7 months compared with 13.8 months for LAPC. There was no statistical significance when comparing either Kaplan–Meier curves (Figure 3C,D).

The ability to move to second line therapy after initial treatment and inability to proceed to resection was evaluated by each group. Of the chemotherapy alone group, 13/26 (50%) were not resected; 11/26 had gemcitabine and nab‐paclitaxel and 2/26 had FOLFIRINOX. Both of the FOLFIRINOX patients were transitioned to gemcitabine and nab‐paclitaxel in the second line. All 11 gemcitabine and nab‐paclitaxel patients were transitioned to chemoradiation after disease progression, deterioration in clinical status, or intolerable side effects. None of the 13 patients that started with concurrent chemoradiation were transitioned to any second line therapy.

## 5 DISCUSSION

Pancreatic ductal adenocarcinoma remains one of the deadliest cancers in the world. Surgical resection remains the only method to achieve long‐term survival or cure. However, only a minority of patients present with non‐metastatic disease, and only a subset of these patients will be surgical candidates depending on blood vessel involvement or surgical contraindications. Despite several observational and randomized studies, there is still no clear consensus as to what a superior neoadjuvant regimen in BRPC or LAPC may be. Our study examines both of these subsets of localized PDAC with three different treatment modalities: (1) chemotherapy alone (2) chemotherapy followed by chemoradiation, and (3) concurrent chemoradiation.

In terms of resection, our study's BRPC population demonstrated a 39% overall resection rate, with 69% of them being R0 resections. Our overall resection rate was lower than the rate reported in several recent meta‐analyses of BRPC, which found an overall resection rate ranging from 65 to 69%. The rate of R0 resection was 54% in Dhir et al's meta‐analysis, which included a variety of neoadjuvant regimens. In Janssen et al's meta‐analysis, which looked solely at neoadjuvant FOLFIRINOX in BRPC, the resection rate was 84%.^10^, ^32^, ^33^

Within the LAPC population, our study showed a 10% resection rate, lower than the 23–28% rate reported in recent meta‐analyses, although, admittedly, our LAPC sample size was small (19 total LAPC patients, of which only two underwent resection).^10^, ^34^

One potential explanation for these discrepancies in resection rate between our study and these meta‐analyses may be the fact that the majority of our patients received a gemcitabine‐based regimen (88%), with a minority receiving FOLFIRINOX (12%) (Table S1A:). Recent retrospective studies comparing gemcitabine and nab‐paclitaxel to FOLFIRINOX in BRPC and LAPC have shown a benefit in favor of FOLFIRINOX in terms of both pathological and clinical parameters, including resection rate, PFS, and OS.^23^, ^25^, ^35^, ^36^, ^37^, ^38^ Another explanation for our cohort's lower resection rates was that the mean age (70 years) of this study is higher than any other study examining chemotherapy versus chemoradiation (Katz et al. 2016: 64 years old, and Katz et al. 2021: 65 years old).^23^, ^24^

In terms of resection rate stratified by neoadjuvant treatment modality, chemotherapy alone had a significantly higher rate of resection at 50% (13/26) compared to chemotherapy followed by chemoradiation 8% (1/13) or concurrent chemoradiation 8% (1/13) (Table 1). Admittedly, two significant limitations to our study are the selection bias and moderate sample size. Patients typically chosen for chemoradiation alone during multidisciplinary tumor board may be selected due to perceived intolerance to combination chemotherapy regimens. Indeed, 13/26 patients who started with upfront chemotherapy were ultimately moved to chemoradiation due to either disease progression or chemotherapy side effects. However, this finding is consistent with a previous meta‐analysis assessing the ability of neoadjuvant chemoradiotherapy to downstage BRPC, which was found to be only 16%.^36^ Additionally, patients needing combination chemotherapy alone to get to surgery have improved ability to attempt R0 resections.^24^

In terms of median PFS and median OS, patients that underwent neoadjuvant chemotherapy alone had the most favorable results (16.4 and 26.2 months, respectively) compared to patients that underwent chemotherapy followed by chemoradiation (14.9 and 13.0 months, respectively), or chemoradiation (7.3 and 6.9 months; *p* < 0.0025 for mOS and *p* < 0.0001 for mPFS) (Figure 3A,B). Notably, there was no statistical significance when comparing overall survival curves between BRPC versus LAPC, suggesting that the above difference in median PFS and median OS is indeed treatment‐dependent (Figure 3C,D). Although the groups in Katz et al. 2021 were not meant to be compared, our data is in line with the finding that patients needing chemotherapy with radiation did not have improve overall survival when compared to patients necessitating only chemotherapy to achieve resection.^24^

The most common chemotherapy regimen used in this cohort was gemcitabine and nab‐paclitaxel and FOLFIRINOX was second (Table S1A:). As noted above, three recent retrospective cohort studies comparing gemcitabine and nab‐paclitaxel to FOLFIRINOX seemingly demonstrate superiority in favor of FOLFIRINOX. However, the randomized, prospective SWOG S1505 trial evaluated perioperative FOLFIRINOX vs gemcitabine with nab‐paclitaxel in the resectable population. The primary endpoint of overall survival at 2 years was non‐significant between the two regimens (FOLFIRINOX: 22.4 vs gemcitabine with nab‐paclitaxel: 23.6 months). Additionally, S1505 found no significant difference between gemcitabine with nab‐paclitaxel and FOLFIRINOX in enabling patients to proceed to resection (70% and 73%, respectively).

Our study does have baseline characteristic differences that may explain the above results, most notably the significantly increased tumor size (Table 1). Further, the chemotherapy alone group had most tumor located in the head of the pancreas compared to chemotherapy with radiation. Pancreas cancer within the head generally permits earlier detection due biliary and pancreatic duct dysfunction.

The ability to move to a second line regimen is important in pancreatic cancer as intolerable front line therapies can subsequently hinder a patients survival. All thirteen patients who started on gemcitabine and nab‐paclitaxel were able to move to concurrent chemoradiation, albeit with only one patient able to get to resection. In the FOLFIRINOX group, the 2 patients unable to achieve resection were capable of moving to gemcitabine and nab‐paclitaxel in the second line. In the concurrent chemoradiation group, none of the 13 patients were able to move to second line therapy. These outcomes, however, more likely align with the patient's performance status and selection bias while less on treatment modality. Most patients selected for combination chemotherapy have a performance status that enables side effect tolerance and this translates into additional lines of therapy. Those patients selected for chemoradiation from the start likely had a lower ability to handle multi‐drug chemotherapy regimens neoadjuvantly as evidenced by the higher mean age of this group (73 years).

Ultimately, our study is in line with recent evidence suggesting that chemotherapy with radiation may not have superior results compared with combination chemotherapy alone in borderline‐resectable and locally‐advanced pancreatic cancer patients. There are important caveats to this statement, however. Experienced chemotherapy, radiation, and surgical centers with multidisciplinary tumor boards exist to parse out borderline‐resectable and down‐staged locally‐advanced patients that would have a higher likelihood of obtaining an R0 resection. The addition of radiation may permit an otherwise difficult surgical procedure to move forward in some cases with the benefit of knowing a close vessel margin will have been radiated preoperatively.^17^

Additionally, the field of intraoperative irreversible electroporation (IRE) during surgical resection is an evolving field that may also extend surgical clearance of tumor in otherwise unresectable patients at some larger centers.^39^ Further, chemotherapy with radiation permits ‘testing’ of the tumor biology and possibility of metastases prior to an intensive surgical resection and recovery. Why some patients respond to chemotherapy with or without radiation treatment is likely a reflection of diverging tumor biology. Studies may select for a population of pancreatic cancer patients whose biology responds well to chemotherapy alone versus a possible non‐responsive biology that may spur the use of radiation. As is true for all cancer types, the field of pancreatic cancer has a great need for the identification of markers that would predict an individual's tumor responsiveness to different chemotherapy modalities as well as radiation therapy.

## 6 LIMITATIONS

The limitations of this study are inherent to any retrospective cohort study, namely lack of randomization leading to potential uncontrolled confounders, lack of blinding potentially influencing treatment choice, and moderate sample sizes in the borderline‐resectable and locally‐advanced pancreatic cancer groups. An important caution to the results of our study is that it was not prospective. As such, our data is confounded by the fact that the choice to use radiation usually occurs after chemotherapy alone does not cause enough tumor regression to allow for surgery. Also, patients are selected for chemoradiation over neoadjuvant chemotherapy when the medical oncologist perceives their performance status to be intolerance of combination chemotherapy. Prospective studies should be able to control this issue with randomization between a chemotherapy alone arm and a chemotherapy with radiation arm prior to resection.

As mentioned above, there are statistically significant differences in baseline characteristics between the groups, specifically tumor size and anatomic location. We acknowledge a lower use of neoadjuvant FOLFIRINOX during the period of 3/1/2014–3/1/2019 compared with gemcitabine with nab‐paclitaxel in this more elderly population. Although no difference was observed in the resectable pancreatic cancer population in the perioperative setting (in line with SWOG 1505), the uneven distribution of patients may have affected our outcomes. Finally, this was a single‐center experience, although in line with recent multi‐institutional studies.

## 7 CONCLUSIONS

BRPC and LAPC patients capable of surgery after only receiving neoadjuvant treatment with chemotherapy had higher rates of R0 resection with prolonged median PFS and OS compared to any patient needing combination chemotherapy with radiotherapy.

## AUTHOR CONTRIBUTIONS

**Tridu R Huynh:** Conceptualization (supporting); data curation (equal); formal analysis (equal); investigation (equal); methodology (equal); project administration (supporting); resources (supporting); writing – original draft (equal); writing – review and editing (equal). **Darren Sigal:** Data curation (equal); formal analysis (equal); supervision (equal). **Randolph Schaffer:** Data curation (equal); supervision (equal); writing – original draft (equal). **Samanta R Spierling‐Bagsic:** Data curation (equal); formal analysis (equal). **Ray Lin:** Data curation (equal); formal analysis (equal); investigation (equal); supervision (equal). **Gregory P Botta:** Conceptualization (lead); data curation (lead); formal analysis (lead); funding acquisition (equal); investigation (equal); methodology (equal); project administration (equal); resources (equal); validation (equal); writing – original draft (equal); writing – review and editing (equal). **Alexander Agelidis:** Data curation (equal); formal analysis (equal); writing – review and editing (equal).

## FUNDING INFORMATION

This work was supported by NIH NCATS CTSA grant KL2TR002552.

(GPB, TH), NIH NCI LRP KYGF9753 (GPB), institute grant from Scripps Clinic (GPB), and NIH NCATS CTSA UL1TR002550 (SRSB).

## CONFLICT OF INTEREST

None.

## Supporting information


Table S1A:

## Data Availability

The University of California San Diego supports the sharing of Research Data to advance public knowledge. In the interest of advancing knowledge, the University expects Principal Investigators to release and share final Research Data for use by other investigators and researchers in a timely manner, consistent with the practices of the discipline involved, and in accordance with existing University policies and guidelines, including those related to intellectual property, sponsor requirements, and applicable laws and regulations, such as laws relating to protecting the rights and privacy of human subjects.
