# Supplementary material for: Neoadjuvant chemotherapy and radiotherapy outcomes in borderline‐resectable and locally‐advanced pancreatic cancer patients
Source: Cancer Med. 2022 Dec 7;12(7):7713–23. doi: 10.1002/cam4.5523 (PMC10134275; doi:10.1002/cam4.5523)
Supplement: Supplementary file 1 — Table S1A: [file CAM4-12-7713-s001.docx]

**Supplemental Table 1A: Chemotherapy Regimen Breakdown**

|  | | All Patients (N=52) | Chemotherapy Alone  (N=26) | Chemotherapy followed by Chemoradiation (N=13) | Concurrent Chemoradiation (N=13) |
| --- | --- | --- | --- | --- | --- |
|  |  | |  |  |  |
| Gemcitabine + Nab-Paclitaxel, N (%) | 32 (62) | | 20 (77) | 12 (92.3) | 0 |
|  |  | |  |  |  |
| FOLFIRINOX, N (%) | 6 (12) | | 5 (19) | 1 (7.7) | 0 |
|  |  | |  |  |  |
| Gemcitabine, N (%) | 14 (26) | | 1 (3.8) | 0 | 13 (100) |

**Supplemental Table 1B: Rates of R0, R1, and Non-Resection by Stage**

|  | Borderline-resectable | Locally-advanced |
| --- | --- | --- |
| R0, N (%) | 9 (28) | 2 (10) |
|  |  |  |
| R1, N (%) | 4 (12) | 0 (0) |
|  |  |  |
| Non-resected, N (%) | 20 (62) | 17 (89) |
